# Africa's largest long-lasting insecticide-treated net producer: lessons from A to Z Textiles

**DOI:** 10.1186/1472-698X-10-S1-S6

**Published:** 2010-12-13

**Authors:** Hassan Masum, Ronak Shah, Karl Schroeder, Abdallah S Daar, Peter A Singer

**Affiliations:** 1 McLaughlin-Rotman Centre for Global Health, University Health Network and University of Toronto, 101 College Street Suite 406, Toronto ON, M5G 1L7, Canada

## Abstract

**Background:**

Field trials have demonstrated the efficacy of insecticide-treated nets, and the WHO has recently endorsed a shift toward Long-Lasting Insecticide Treated nets (LLINs) due to factors such as reduced distribution costs. However, the need for LLINs poses several challenges. Is it possible to manufacture LLINs in large quantities in the African continent, where malaria is most endemic? When production is located in low-income countries, what role is played by local funding and employment, scaling up manufacturing, and partnerships? What factors influence availability and pricing?

**Discussion:**

A case study of A to Z Textiles was undertaken to answer the question of how large-scale production of LLINs can occur in a low income setting. One of the largest sources of bed nets for Africa, A to Z Textiles is Africa-based, and its Tanzanian operations have a production capacity of 30 million LLINs per year, along with full WHO recommendation for its nets. Our analysis is based on semi-structured interviews with key informants familiar with A to Z, site visits in Tanzania, and literature reviews.

This paper discusses the history and current status of A to Z Textiles, identifies the factors that led to its success, and suggests policy considerations that could support similar initiatives in the future. Local funding, scaling up manufacturing, technology transfer, and partnerships all played important roles in A to Z’s ascent, as did perceived benefits of local employment and capacity-building. Regulatory issues and procurement rules acted as barriers. A to Z cost-effectively manufactures high-quality LLINs where malaria is most endemic.

**Summary:**

With a production capacity of 30 million LLINs per year, and full WHOPES (WHO Pesticide Evaluation Scheme) certification, A to Z Textiles demonstrates how key health goods can be successfully produced in the low-income countries that use them. Its example may be instructive and of high interest to readers in the malaria community, especially in developing countries, and to those who wish to support or partner with efforts by developing countries to build their health innovation capacity.

## Background

Most of the malaria disease burden is in Africa, with a cost in human life of around one million deaths per year, of which a large majority are children [1]. Among several approaches such as vector control strategies that are being pursued to prevent malaria, insecticide-treated nets (ITNs) are one of the best current public health interventions in terms of safety and cost-effectiveness [1]. Large-scale studies have documented the beneficial impact of ITNs on reducing malaria mortality and morbidity, including infant mortality [2,3].

More recently, a shift toward Long-Lasting Insecticide-treated Nets (LLINs) has been endorsed by the WHO Global Malaria Programme (WHO/GMP) [4]. LLINs are made with material that has insecticide bound around or incorporated within the fibers. They must retain effectiveness for at least 20 WHO standard washes (i.e. laboratory procedures that simulate usage) and three years of use in field conditions. LLINs reduce the need for retreatment and replacement of nets, offer longer protection for users, and help reduce distribution and maintenance costs.

While the reasons for preferring LLINs are clear, the production of LLINs poses several challenges. Is it possible to manufacture them in large quantities in the African continent, where malaria is most endemic? When production is located in low-income countries, what role is played by local funding and employment, scaling up manufacturing, and partnerships? How do regulatory issues and procurement rules affect the viability of LLIN production? What factors influence availability and pricing?

These issues are illustrated in the case of Africa's largest bed net manufacturer, A to Z Textiles – a family-based Tanzanian enterprise that grew from humble beginnings to become a major bed net manufacturer. A to Z illustrates the role of African entrepreneurship in successful technology transfer and development. It shows how supportive partnerships can mobilize the South’s capacity to tackle its own health challenges.

We used a case study design. Our analysis is based on semi-structured interviews with key informants familiar with A to Z, site visits in Tanzania, and literature analysis of background documents on A to Z, related articles from the peer-reviewed literature, news reports, books, web sites, and relevant reports from WHO (the World Health Organization) and other groups. We conducted interviews with personnel of A to Z, and with PSI (Population Services International), Acumen Fund, and Tanzanian government and health organizations, all of whom interacted with A to Z in its bed net endeavors. A to Z was asked to fact-check the case study; the analysis and interpretation is our own. All quotes are from the interviews unless otherwise noted, and with permission. This study was approved by the Office of Research Ethics of the University of Toronto.

In this article, we summarize the history and current status of A to Z Textiles, and analyze the factors that led to its success. We then suggest policy considerations that could further support similar producers of health goods in low income settings in the future.

## Discussion

### A chance meeting

A to Z Textiles is based in Arusha, Tanzania. The company started in 1965 with five sewing machines run by family members of the late N.H. Shah (father of the current CEO Anuj Shah). The family initially made garments for the local market, and in 1978 they started producing bed nets.

In 1998, Professor Don De Savigny, then with Canada’s International Development Research Centre, was visiting Arusha with Dr. Hassan Mshinda, then director of Tanzania’s Ifakara Health Research Institute. They had come to meet the owners of Sun Flag, a local company that made bed nets.

While insecticide-treated bed nets were not new, they had a significant problem: they needed to be regularly re-dosed with insecticide. The companies that made the insecticide preferred to sell it by the barrel, and the consumers who bought the nets often had more urgent priorities than bringing the nets to a central depot to be re-treated. De Savigny hoped Sun Flag would partner with an initiative from PSI (Population Services International, a non governmental organization with global operations) to provide home treatment kits that people could use to dose the nets themselves.

Sun Flag wasn’t interested, and De Savigny and Mshinda found themselves back on the street. Dr. Mshinda pointed out that there was another bed net manufacturer across the road: a 33-year old family-run company called A to Z Textiles. With nothing to lose, the two men knocked on the gate at A to Z. They were met by Anuj Shah and his Production Director, Binesh Haria.

### Opportunity and pitch

The timing of De Savigny and Mshinda’s visit might not have been right for Sun Flag, but it was perfect for A to Z. De Savigny and Mshinda were looking for ways to alleviate the suffering caused by malaria, and when they pitched the home treatment kit as a business opportunity, Anuj Shah immediately saw its potential.

By the mid 1990s, their business had been facing challenges for years because trade liberalization in the 1980s had resulted in a large influx of cheaper second hand clothing into the local Tanzanian garments market. A to Z already made bed nets, but neither Anuj nor Binesh had guessed how important nets could be in the fight against malaria.

According to De Savigny, he told Anuj Shah, “If you get into this, you could steal the margin on this, because the public sector is about to start promoting this product.” He pointed out that public sector institutions were never going to promote A to Z’s baseball caps or T-shirts, “but they will promote this one, and if you have a better net [than Sun Flag], you’re going to get the business.”

This first meeting with A to Z was cordial, but as De Savigny put it, “We thought, well, it’s another one of these factories and they’re not going to listen to us…it was a very polite meeting and we got their attention, but we didn’t think too much about this afterward.” De Savigny and Mshinda left Arusha to continue promoting the home kits for re-dosing ITNs elsewhere. Six months after that first encounter, a meeting was held in Dar es Salaam by Canada’s International Development Research Centre, PSI, and Health Bridge Canada. The purpose of the meeting was to bring the public sector, private sector, and NGOs together to explore ways to engage both the public and private sectors in rolling out ITNs across Tanzania. A to Z was invited to the meeting, but no one expected them to come. Professor De Savigny describes what happened:

“We had a reception to kick off the meeting and we had the Chamber of Commerce, the various Ministries, and the high level officials from the Ministry of Health… we made an exhibition space in the reception area for the insecticide manufacturers [who were] by then making kits, and for any net manufacturers who wanted to show up. So Sun Flag came and they hung up their standard white conical net on this white wall. And just before the speeches started, in came Binesh and Anuj. As we said, we never thought we’d see these people again, and they walked in and they hung up their very first six foot by six foot green, silky rectangular net – [a] huge net, and they hung it up on the wall and everybody flocked over there to see this fantastic new, domestic net. AMREF, the African Medical Research Foundation, was there from Nairobi and they went up and they gave an order for 100,000 on the spot.”

### From Arusha to Tokyo

By 2003 A to Z was the largest producer of conventional (i.e. non long-lasting) insecticide impregnated nets in Africa, producing over 6 million nets annually. This success was enabled by A to Z’s strengths in plastics, textiles, and production technologies, which positioned A to Z to scale up production and adopt new technologies when Sumitomo Chemical Company of Japan presented them with an even bigger opportunity.

Sumitomo was founded in 1913, and has grown to be a multinational organization with over 100 companies in sectors including basic chemicals, petrochemicals, fine chemicals, and pharmaceuticals. Since the 1980s, scientists at Sumitomo had been developing insect-repellent products. Their LLINs had insecticide incorporated into the fiber of the net, which allowed them to release insecticide for up to 5 years. Primarily a chemical company, Sumitomo lacked expertise in packaging, marketing, and distribution in the developing world, and was searching for a partner.

In 2002 the World Health Organization (WHO) and the Roll Back Malaria Partnership approached Sumitomo, encouraging the company to look into manufacturing “Olyset” LLINs in Africa. A to Z was one company short-listed by WHO for Sumitomo to visit, and was deemed an attractive candidate with expertise in both textiles and plastics.

The dynamic synergy between A to Z Textiles and Sumitomo led to a royalty-free technology transfer of Olyset LLIN technology to A to Z. The technology transfer was facilitated by WHO, the social venture capital group Acumen Fund, and the Roll Back Malaria Partnership [5]. Dr. Mshinda further explains the terms of the transfer:

“The technology transfer entails that the raw material with the insecticide inside is provided by Sumitomo in its pellet form, along with their expertise around the technology. A to Z’s expertise lies in manufacturing this product. Sumitomo is basically a chemical company, so they do not have that expertise in manufacturing. The actual process of incorporating the insecticide remains with Sumitomo, which is a [trade] secret.”

### A to Z today: capacity and clinical evidence

The technology transfer for Olyset bed nets was completed in November 2003, heralding the start of the business partnership between Sumitomo and A to Z Textiles. They began with a trial run of 300 000 LLINS in 2003, reached 1.5 million in 2005, and by 2009 production had grown to 25 million Olyset bed nets per year.

A to Z also produces textiles, plastics, and bottled water. In 2009, LLINs accounted for roughly 80% of total revenue and 90-95% of production. The joint venture with Sumitomo dedicated to Olyset production was set up in 2005 as an affiliated company, Vector Health International, in which Sumitomo and A to Z reportedly made equal initial co-investments of $7.5 million.

As of 2010, A to Z was a profitable enterprise employing over 7000 staff. Their annual production of 25 million nets was projected to increase to 30 million nets during 2010. Only 5% of A to Z’s output goes to private buyers, mainly the middle and upper class. A to Z’s biggest non-private buyers are organizations like UNICEF, and the governments of Tanzania, Mozambique, Malawi and Kenya, which have a variety of distribution models and subsidization schemes to deliver nets to end users.

A to Z anticipates a total need of around 280 million bed nets in Africa over the next three years; i.e. a market demand of approximately 85 million nets per year. Of these, A to Z is projected to produce roughly 30 million Olyset nets annually in partnership with Sumitomo, and Sumitomo is projected to produce an additional 21 million Olyset nets in facilities based in China and Vietnam.

By way of comparison, according to the World Malaria Report 2009, in 2008 manufacturers cumulatively reported a total of 57 million bed nets delivered to African countries, and ministries of health cumulatively reported a total of 38 million bed nets distributed within African countries [6]. (The report suggests that the difference between these two figures could be due to the lag between delivery and distribution, inadequate record-keeping, distribution by the private sector that is not included in ministry of health data, or other unknown factors.)

The Olyset net was the first LLIN to gain full WHO recommendation under the WHO Pesticide Evaluation Scheme (WHOPES), in October 2001 [7]. It was one of only two LLINs to have achieved full WHO recommendation as of August 2009, the other being the Vestergaard Frandsen Permanet 2.0 [8]. (The latter was reported in company documents to be manufactured in Vietnam and Thailand, and have a cumulative total of over 170 million distributed worldwide.) Olyset is the only LLIN with full WHO recommendation being manufactured in Africa.

There is evidence that Olyset nets retain some efficacy even after seven years in the field [9,10]. However, after a review of available evidence in 2009, the WHOPES Working Group decided not to extend WHO full recommendation from 3 to 5 years [11], indicating that the evidence could not prove that sufficient efficacy was maintained in the 4^th^ and 5^th^ years.

We turn now to a discussion of what helped A to Z succeed, and what their example suggests for future development of health solutions in low-resource settings.

### Lessons learned

#### Family funding and entrepreneurship

A to Z’s holdings are primarily family-owned – the Shah family has invested millions of dollars, making them the biggest investor along with Sumitomo. Family financing is a common formula in African and South Asian business ventures, especially when alternative risk capital is absent, as it can provide both capital and “smart money” that knows what ventures are feasible in the local environment [12]. While family financing holds long-term risks from infighting, loss of competence, and corruption, evidence of this was not found in the case of A to Z, and family firms dominate in most countries with short industrial histories [13].

The fact that the Shahs chose to take the risk of investing in LLIN production was itself a key decision point; the chance meeting with De Savigny and Mshinda in 1998 was key to making this opportunity apparent. Similarly, the Sumitomo partnership for Olyset production was a second key decision point, in which several partners assisted [5]. These two decision points may illustrate the potential benefit to future funders and entrepreneurs of “global health market intelligence” that makes key opportunities more apparent.

#### Manufacturing at scale and partnerships

A to Z has been instrumental in bringing manufacturing to scale in Tanzania while addressing an important health problem. There appear to be three main reasons why A to Z has been able to go from manufacturing 300 000 LLINs per year in 2003 to 25 million per year in 2009, with a projected 30 million per year in 2010.

First, by the time it had begun discussions with Roll Back Malaria and Sumitomo around LLINs, it was already the largest African manufacturer of ITN nets, producing over 6 million nets by 2003. This prior manufacturing experience was critical in navigating the 2004 Olyset manufacturing ramp-up.

Secondly, A to Z had developed a strong partnership with Sumitomo that not only provided the technology, but also provided financial support through their joint venture. Without the Sumitomo partnership and LLIN technology, A to Z would have experienced difficulty in achieving WHO certification and consequent sales; however, without A to Z’s strengths in distribution and local manufacturing, Sumitomo likely could not have achieved the African market success it has. Several parties were involved in making A to Z’s partnerships happen, including WHO, PSI, and Acumen Fund [5]. A to Z itself understood and exploited potential partnerships, most notably in the partnership with Sumitomo for Olyset production. Such relationships can provide incentives and de-risking for the private sector in the developing world to engage in innovation and address health challenges, by making opportunities clearer and by bringing together organizations with complementary capacities. (Indeed, there may be value to doing this sort of matchmaking more systematically – both for Southern enterprises looking to get into the health field, and for Northern organizations wishing to find capable and reliable Southern partners [14].)

Finally, A to Z benefited from commitment and large demand from buyers, mainly the donor community. About 95% of production goes to non-private buyers, such as UNICEF, the Global Fund to Fight AIDS, Malaria and Tuberculosis, the President’s Malaria Initiative, PSI, and African governments. In the absence of donor and public funding, i.e. relying solely on local private buyers, A to Z would have had to lower volume sold, pricing, or both – suggesting the catalytic role that donor and public funding can play in scaling up Southern manufacturing. It remains to be seen whether local demand (both public and private) will supplant international funding over time. To support similar future ventures, it may be helpful to study the de-risking role to producers of commitments from public buyers made in advance of production, in order to entice the private sector into areas of public health they would normally not enter [15].

#### Distribution and marketing

A to Z has used three linked distribution channels. First, a traditional private distribution network, done through an extensive network of wholesalers and retailers in eastern and southern Africa. Second, a mobile distribution system composed of a fleet of 150 trucks within Tanzania, which is supplemented by trucks hired from transport companies if demand warrants, and reaches countries surrounding Tanzania including Kenya, Uganda, Zambia, Zimbabwe, Mozambique, Burundi, and Rwanda. (One 40-foot container holds 26 000 nets.)

Third, for their public sector buyers, A to Z delivers the nets to designated warehouses or the appropriate Ministries of Health, building on their private and mobile distribution systems. This represents a significant advantage over their international competitors, who may only deliver to national ports. Looking ahead, there may be opportunities for manufacturers like A to Z to expand their logistics support to public sector buyers and NGOs, in areas such as training users or monitoring bednet replacement statistics.

#### Regulation and tendering

Being one of only two LLINs to gain full WHO approval has made Olyset a preferred product for buyers. At the same time, Anuj Shah notes that some tenders for bednets require only interim WHO certification, for which there are more competitors (six as of August 2009) [8]. Even though bednets with the full certification are supported by a greater evidence base from field trials, this distinction has not been implemented by some buyers in the tendering process, which may put A to Z and other fully-certified manufacturers at a competitive disadvantage if they invest more in quality.

Some tenders for LLINs evaluate bids only on the cost to the port of shipping. Anuj Shah suggests requiring full certification in tenders, and using the price to village as the tender price: “I think the responsibility of delivering the nets to the village should be on the manufacturers. Donor should say that you deliver. How you deliver is up to you.” Using “full-cost” pricing could be a win-win situation, with buyers and funders able to evaluate long-term pricing for the provision of malaria protection to end users, and manufacturers able to invest in building up quality and distribution expertise.

#### Looking forward: need for innovation

Looking forward, A to Z faces the prospect of market saturation and a plateau in demand for their bed nets in the 2011 to 2013 time frame. In addition, other African LLIN manufacturing partnerships have recently emerged. Duranet, an LLIN with interim WHO certification, has been manufactured in Uganda on a low-volume trial basis starting in December 2009, in co-operation with two local companies [16]. AED, Bayer Environmental Science, and Siamdutch Mosquito Netting Company of Thailand developed a system treating finished nets with LLIN chemistry; this system was reportedly installed by Sunflag/Nigeria in 2009 [17]. Sumitomo itself has in 2009 established an Olyset net sewing operation in Ethiopia, in a joint venture with an Ethiopian company with a projected production of 3 million in its first year; Sumitomo has also stated its intention to build a Nigerian factory [18].

One response to these challenges that A to Z is exploring is increased investment in R&D, including adding laboratory facilities and local scientific talent. A to Z has already hired one full time scientist who used to work with WHO. Having benefited from one technology – indeed, having integrated a culture of technology into their business – they now seek to identify and exploit other opportunities, including potential linkages with domestic scientists and further partnerships with companies like Sumitomo.

In combination with R&D innovation, A to Z faces pressure to continue its business model innovation, through means such as further developing delivery capabilities, and improving packaging and user training. Other innovations may straddle R&D and business, such as cost reduction and more effective bednet efficacy maintenance. While continuous innovation and improvement is a linchpin of business globally, A to Z’s experience illustrates the importance of an integrated view of innovation covering technical, business, and social aspects.

A reflection from Anuj Shah illustrates the management’s acceptance of both economic and health goals:

…My biggest motivation is the number of lives we are saving. That is a real motivation. That’s one. Number two – Africa needs jobs and without people getting jobs, this continent will never develop. The more jobs that are created gives me personal satisfaction that at least people are having an activity, they’re earning out of it and they are benefiting out of it.

Of course the company is growing so that is really something very, very important for me and really motivates me. Third thing – when I wake up, it makes me feel that we have to make sure that we have enough orders for the [7000] people to have enough work to carry on, so that itself is a challenge and also to some extent it’s a motivation. It always puts you on the edge; we just need to get an order. If you don’t have the orders, where are these people going to be sleeping, what are they going to be eating? So that is a real motivation or I can say challenge.

I sometimes wish we had more than 24 hours in a day.

## Summary

The A to Z story shows that high-quality LLINs can be cost-effectively manufactured in Africa, where malaria is most endemic. This has been achieved without tariffs or other protectionist measures that might lead to significantly higher-priced local ITN production [19]. It demonstrates that health products can be developed by leveraging existing strengths in low-resource settings, leading to health, economic, and technological benefits for these regions. Many such opportunities have been documented in the African context, such as manufacturing common medical supplies [20].

Ultimately, success is enabled by responsiveness to opportunities, willingness to invest and take risks, ability to execute, and strong leadership. A to Z and its partners have not only successfully created an African source for public health goods, but have demonstrated consistent commitment to the endeavor. In the absence of the private enterprise A to Z, it is doubtful that any local public entity would be producing LLINs at the same scale.

Donor funding for bednets played a critical role in creating demand for A to Z’s bednets, suggesting that donor and multilateral agencies can kickstart Southern manufacturing by integrating such enterprises into their supply chain. Local funding, scaling up manufacturing, technology transfer, and partnerships with Sumitomo and others all played important roles in A to Z’s ascent, as did perceived benefits of local employment and capacity-building; regulatory issues and procurement rules acted as barriers.

Yet continued success is not assured – the looming plateau in demand for LLINs, competition from imported LLINs sourced from Asian manufacturers, and the recent founding of multiple African sites for LLIN manufacturing all suggest the need for continued innovation to stay in business. In this and other aspects, A to Z is a microcosm of the African-led health innovation enterprise as a whole.

## Competing interests

The authors declare that they have no competing interests.

## Authors' contributions

HM, ASD, and PAS conceived the study. RS, ASD, and PAS carried out fieldwork. HM, RS, and KS carried out the analysis. HM, RS, KS, ASD, and PAS drafted the manuscript. All authors read and approved the final manuscript.
